# Mental Symptoms and Patients' Thoughts

**Published:** 1914-06-13

**Authors:** 


### MENTAL SYMPTOMS AND PATIENTS' THOUGHTS.

### The Methods of Psycho-analysis.

The attention that has lately been focussed on the process of psycho-analysis in the treatment of mental and nervous disorders owes its inception, as pointed out in a previous article (The Hospital, May 16, 1914), to the painstaking researches of Professor Sigmund Freud and his school. As the term indicates, psycho-analysis is the detailed examination of a patient's mental life with the object of resolving as far as possible the various trains of thought which appear to have a bearing on the particular neurosis or psychoneurosis for which relief is sought. Clearly, every physician who interrogates an invalid in regard to mental symptoms obviously carries out, so far as he can, an analysis of the patient's thoughts, but such an investigation is limited in its scope by the sufferer's memory as well as by his not infrequent loss of attentive power. It is also limited indirectly by the examiner's want of knowledge as to what line his conversation should follow. Consequently, a need has for some time been felt for measures auxiliary to these ordinary mental analyses of the consulting-room, and it is because the method of psycho-analysis has seemed likely to be of vast assistance in this connection that so many workers have eagerly taken it up. The whole point of the process about to be detailed rests, of course, on the assumption that the bringing to light of certain suppressed ideas or the tracing to its original source of a morbid train of thought, will place us in a position to remove by one means or another, with which we are not now concerned, any particular mental or nervous trouble. In practice the analysis is at the present time commonly carried out in either of two ways, one of which is termed "free association," and the other "word association."

### Free Association.

When it is desired to carry out psycho-analysis according to the first plan the patient is asked to take up a restful position and give his whole attention to the matter in hand; that is to say, to keep in mind the fact that he is being examined mentally with reference to some particular symptom. After a minute or two the medico-psychologist asks him to relate whatever thoughts happen to pass through his mind, so that the invalid begins a sort of running monologue in which a host of ideas is revealed. No matter how trivial, how absurd, or how irrelevant the thoughts that come to the patient may be, they are to be mentioned in due sequence and with proper care. Whilst this is going on, the physician takes note in writing, as far as possible, of what the patient says. Whenever something is mentioned that appears to have a bearing on the illness which is being dealt with, the examiner makes a special note of it, and may, if he likes, turn the patient's thoughts in special directions by asking a question or by giving a hint here and there. Having carried on this novel form of examination for as long as he considers sufficient, the investigator proceeds to the interpretation of his material; and this is the most difficult part of the whole business. Space precludes any detailed account of the elaborate methods of interpretation which have been devised by the pioneers of psycho-analysis, as will be understood when it is remembered that their theories have been built up on countless experiments extending over some twenty or thirty years. Suffice it to say that interpretation depends on reference to various "thought symbols" the exact meaning of which has in some instances been determined by psychological experiments.

### Word Association.

Another way of arriving at a similar end is by telling the patient to pay attention not to his train of thoughts, but to ideas called up by various words which are read out to him by the examiner. The patient is seated in a comfortable chair, preferably with his back to the doctor, and it is the examiner's duty when following this plan of analysis to note certain reactions obtained in response to various spoken words. The first and foremost of these is a time-reaction, and depends on the time which elapses between the test-word and the patient's response. This is carefully measured by a stop-watch in fifths of seconds. When someone calls out a word and another person is asked to say the first word that comes into his mind afterwards, the response is usually quick; not more than four or five fifths of a second elapsing between the test-word and the reaction. When the reaction is unduly prolonged—say up to 20, 30, or 40 fifths of a second, the analyst makes a note, because it is part of the theory that the test-word and particularly the response-word under such circumstances are associated with a hidden suppressed emotion or idea, that has something to do with the origin of the illness for which the analysis is being carried out. Another important point is the character of the response-word, and here again experience has enabled those who are experts in the method to draw certain conclusions from certain response-words. A British worker whose researches have greatly added to our knowledge of psycho-analysis is Dr. Ernest Jones, of London and Toronto, whose book on the subject has done much to make the new method accessible to English-speaking practitioners.

Other well-known psychologists who have been experimenting with psycho-analysis in this country include Dr. David Forsyth, of Charing Cross Hospital; Dr. Bernard Hart, of University College Hospital; and Dr. Hugh Wingfield. Ernest Jones considers that in addition to the reaction-time and the nature of the response-word amongst these, the following reactions are very important:—

(1) Complete failure of response. A convention limit of thirty seconds is advised; if no response is made before this time it is to be concluded that the test-word has called up some painful or other emotional idea that is important in regard to the analysis. (2) Repetition of the same response-word. (3) Inability to reproduce response-words a short time after they have been spoken. It must be noted that part of the test consists in asking the patient at the end of the examination to repeat from memory the response-words he has given; particular notice is made where he is unable to do so, and in the specimen table given below the sign + signifies accurate reproduction.

Any list of words may be chosen for the purpose of this test, but by experience the psycho-analyst knows that certain words which are likely to be associated with common emotional states are of the greatest assistance to him in his methods. The following table gives an example of a small part of a test actually carried out, although its interpretation and relation to the particular case then dealt with is too lengthy a matter to discuss now.

| Word | Time | Reaction | Reproduction |
|---|---|---|---|
| Fish | 11 | Boiled | + |
| Sun | 9 | Hot | + |
| Cough | 9 | Violent | + |
| Wish | 10 | Hearty | + |
| Thunder | 7 | Loud | + |
| Web | 11 | Web ? Spider, Spider-web | + |
| Talk | 14 | Casual | + |
| Fly ... | 15 | Horsefly | + |
| Dance | 19 | Fancy | + |
| Wear | 18 | Underwear | + |
| Bush | 14 | Flower | + |
| Run | 13 | Hot | + |
| Spite | 75 | Sheer | + |

*N.B.*—It will be noted that the last word given above was followed by an excessive reaction-time—namely, 75 fifths of a second. Subsequently examination proved that the emotional feeling associated with spite had an important bearing on the patient's mental illness.

